# Does quercetin affect tendon healing? An experimental study in a rat model of Achilles tendon injury

**DOI:** 10.3389/fmed.2025.1522517

**Published:** 2025-02-05

**Authors:** Ahmet Yurteri, Numan Mercan, Zeliha Esin Çelik, Hakan Yaykaşlı, Ahmet Yıldırım

**Affiliations:** ^1^Department of Orthopedics and Traumatology, Konya City Hospital, Konya, Türkiye; ^2^Department of Orthopedics and Traumatology, Kahramanmaraş Necip Fazıl City Hospital, Kahramanmaraş, Türkiye; ^3^Department of Medical Pathology, Selçuk University, Konya, Türkiye; ^4^Department of Electronics, Kahramanmaraş İstiklal University, Kahramanmaraş, Türkiye

**Keywords:** biomechanical, histopathological, immunohistochemical, polyphenols, quercetin

## Abstract

**Purpose:**

The objective of this study was to investigate the impact of quercetin, a potent antioxidant, on tendon healing utilizing a rat Achilles tendon injury model.

**Materials and methods:**

The study involved 32 male Wistar-Albino rats, randomly split into experimental (quercetin) and control groups, each with 16 rats. A bilateral Achilles tenotomy model was applied, with the experimental group receiving quercetin and the control group receiving corn oil via oral gavage from surgery until sacrifice. One Achilles tendon per rat underwent histopathological and immunohistochemical evaluations, while the other underwent biomechanical analysis.

**Results:**

Tendons were evaluated histopathologically in terms of tenocyte, ground substance, collagen, and vascularity, and quercetin was observed to significantly increase tendon healing in the experimental group (*p*-values = 0.0232, 0.0128, 0.0272, 0.0307, respectively). In the immunohistochemical analysis, type I collagen, type III collagen, alpha smooth muscle actin (SMA), and Galectin-3 were evaluated, and it was observed that quercetin increased tendon healing (*p*-values = 0.0166, 0.0036, 0.0323, 0.0295, respectively). In the biomechanical analysis, the rupture strength was evaluated with six parameters (failure load, maximum energy, displacement, stiffness, ultimate stress, and strain), and it was observed that quercetin significantly increased the rupture strength (*p*-values = 0.032, 0.014, 0.026, 0.025, 0.045, 0.012, respectively).

**Conclusion:**

Quercetin significantly enhanced tendon healing both biomechanically and immunohistochemically. However, further clinical studies are needed to understand its effects on human tendon healing, as this is the first study of its kind.

## 1 Introduction

Tendon injuries, particularly those affecting the Achilles tendon, are on the rise due to the growing interest in sports and the increasing average lifespan (1–4). However, the healing process for injured tendons tends to be prolonged compared to other tissues, primarily due to factors such as low blood perfusion and slow regeneration rates (5). Consequently, there has been considerable research interest in exploring the effects of different antioxidants or anti-inflammatory agents on this challenging and protracted healing process (6–8).

Quercetin, a member of the flavonoid polyphenol class, is commonly present in nature and in the human diet. Its pharmacological properties, including cytoprotective and genoprotective effects such as anti-inflammatory, antioxidative, and antitumoral activities, have been extensively documented (9). Recent research has particularly emphasized its radical scavenging properties and potent antioxidative effects (10).

Quercetin’s antioxidative activities have been extensively studied and demonstrated through various pathways, both *in vivo* and *in vitro* (11–21). Firstly, it exerts antioxidative effects by directly neutralizing free radicals, thereby reducing potential damage to important biological components such as proteins, DNA, and lipids (22–24). Secondly, it enhances cellular protection against oxidative damage and regulates antioxidative enzymes like superoxide dismutase (SOD) and catalase (25). Additionally, quercetin can chelate metal ions, offering another mechanism to shield against oxidative damage (26). At the cellular level, studies in animal models have shown that quercetin inhibits the activation of inflammatory signaling pathways, notably MAPKs and NF-κB, in response to oxidative stress. Furthermore, it suppresses the formation of proinflammatory cytokines and chemokines (27–32). These findings underscore quercetin’s multifaceted antioxidative properties and its potential as a therapeutic agent in combating oxidative stress-related disorders.

In such studies, it is imperative to comprehend the impact of the techniques and substances employed in both the experimental and control groups on tendon healing and the mechanical properties of the tendon. Numerous studies in the literature have investigated the mechanical properties exhibited by tendons (33–35). Given that tendons are predominantly composed of an extracellular matrix, alterations in biomechanical properties serve as indicators of the healing process (36).

The bioavailability of quercetin from dietary sources typically ranges from 1 to 10%, but supplementation can increase this up to 50%, thereby enhancing its antioxidative activity (11, 37, 38). Although there are a limited number of studies on the effect of quercetin on tendon tissue (10, 39, 40), there is no study in the literature examining its effect on tendon healing, and its role in this process has not been fully elucidated. Our hypothesis posits that quercetin, as a potent antioxidative, may exert positive effects on tendon healing in rats, and our aim is to demonstrate this on an Achilles tendon injury model.

## 2 Materials and methods

Thirty-two male Wistar-Albino rats with an average weight of 487 g (450–525 g) and an average age of 12 weeks were utilized in this study. The rats were obtained from Selcuk University Experimental Medicine Research and Application Center (Konya, Turkey). All surgical procedures were conducted in accordance with the approval granted by the Selcuk University Experimental Medicine Research and Application Center Ethics Committee (decision number 2020/58). Power analysis indicated that a minimum of 14 rats per group were necessary to achieve statistical significance, with a predicted type 1 error of 0.05 and an efficacy power of 0.80. Environmental conditions were carefully controlled, with a temperature range of 21°C ± 2°C, humidity maintained at 55% ± 10%, lighting set at 350 lux (measured at bench level), and a 12∶12 light:dark cycle, with lights on at 0700 and off at 1900. The rats were housed in pairs in cages measuring 595 × 380 × 200 mm (Techniplast UK, 1354G Eurostandard Type IV). Throughout the study, measures were taken to ensure the animals did not experience pain, and adherence to the principles outlined in the Declaration of Helsinki was strictly observed. Two groups, comprising 16 rats each (the quercetin group and the control group), were established for histopathological, immunohistochemical, and biomechanical analyses of tendon healing. Bilateral Achilles tendon injury models were applied to all rats.

### 2.1 Surgical technique

All surgical procedures performed on the rats were conducted under general anesthesia, ensuring the absence of pedal reflex. An intraperitoneal injection of 80 mg/kg ketamine hydrochloride (Narkamon 50 mg/mL, BIOVETA, Czechia) and 10 mg/kg xylazine (Xylazinbio 2% BIOVETA, Czechia) was administered for anesthesia induction. Furthermore, the rats were continuously monitored for pain sensation throughout the procedure, and additional anesthesia doses were administered as needed to maintain adequate pain control. Prior to incision, the surgical areas were shaved and cleansed with povidone-iodine (Batix^®^, Denizpharma, Istanbul, Turkey), and all surgical procedures were carried out under sterile conditions.

**Figure 1:**
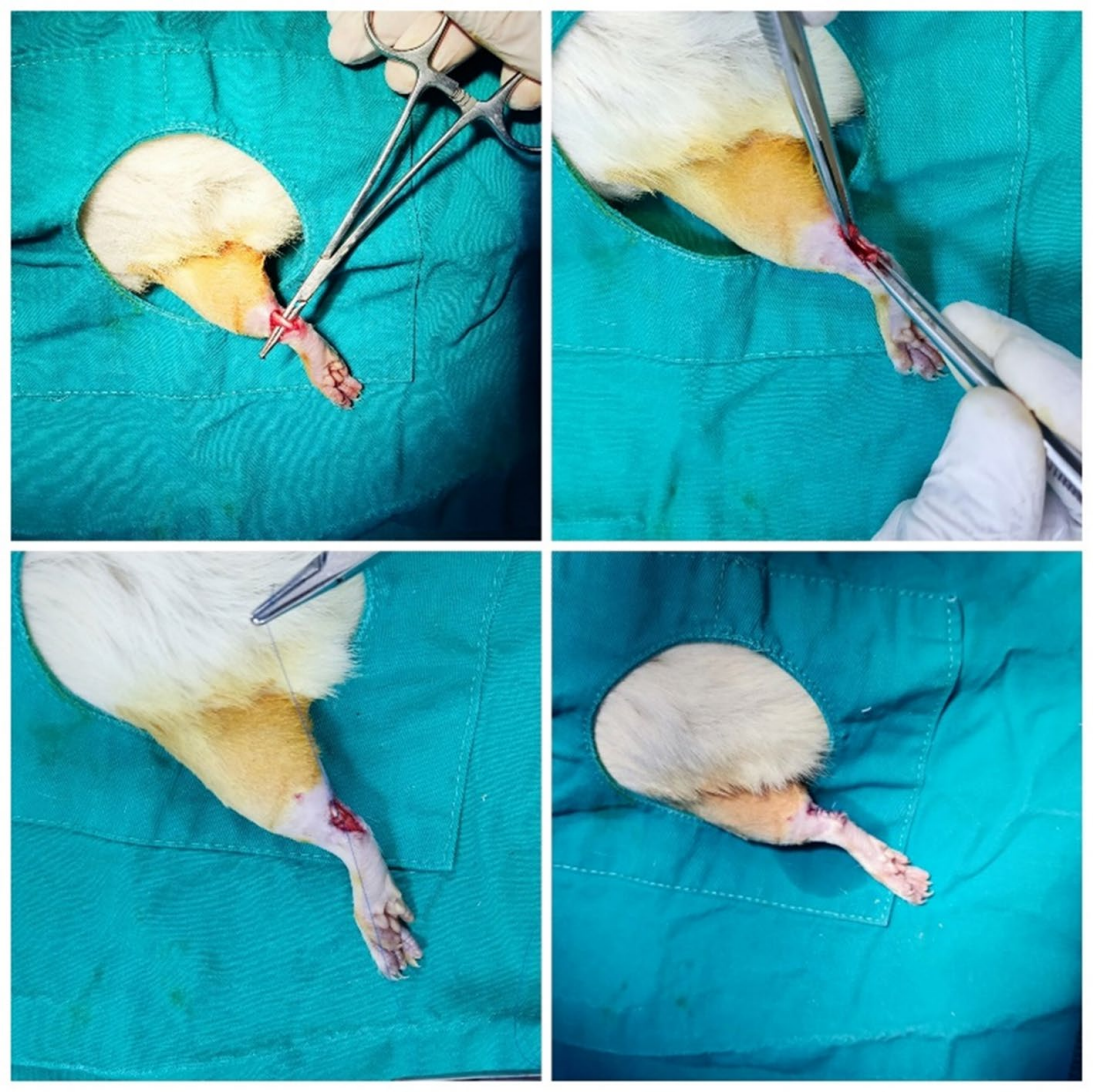
The Achilles tendon injury model and its repair with modified Kessler method.

A well-established tendon injury model, widely utilized in numerous studies in the literature (6, 41, 42), was employed in this study. Following the surgical preparation of the extremity, an incision was made along the posterior line of the ankle to expose the Achilles tendon and plantaris tendon. Tenotomy was performed approximately 0,5 cm proximal to the calcaneus insertion of the Achilles tendon using a No. 15 scalpel blade. The tendons were then repaired end-to-end using the modified Kessler method with 4/0 round polypropylene monofilament sutures (TıpKimSan Limited Company, Istanbul, Türkiye). Following irrigation of the wound area with saline, the incision was closed using 3/0 polypropylene monofilament sutures (TıpKimSan Limited Company, Istanbul, Türkiye) to achieve skin integrity. The surgical procedure is illustrated in Figure 1.

No bandages, dressings, or plasters were applied to the rats postoperatively. From the early postoperative period, all rats were allowed free joint movements and were permitted to load their lower extremities. Rats in the quercetin group received a daily dose of 100 mg/kg quercetin (Sigma-Aldrich, United States), administered as a suspension in corn oil via oral gavage at the same time each day. The control group received only corn oil via oral gavage. All rats were sacrificed on the 28th day following surgery. The quercetin dosage was adjusted based on previous studies (10, 43).

Following administration of high-dose anesthesia (ketamine 75 mg/kg and xylazine 10 mg/kg), rats were euthanized by cervical dislocation. The Achilles tendons of the euthanized rats were then carefully dissected, ensuring removal distally from the bone-tendon junction of the calcaneus and proximally from the bone-tendon junctions of the femur and tibia. Subsequently, one of the Achilles tendons from each rat underwent histopathological and immunohistochemical evaluation, while the other tendon was subjected to biomechanical analysis.

### 2.2 Histopathological analysis

**Figure 2:**
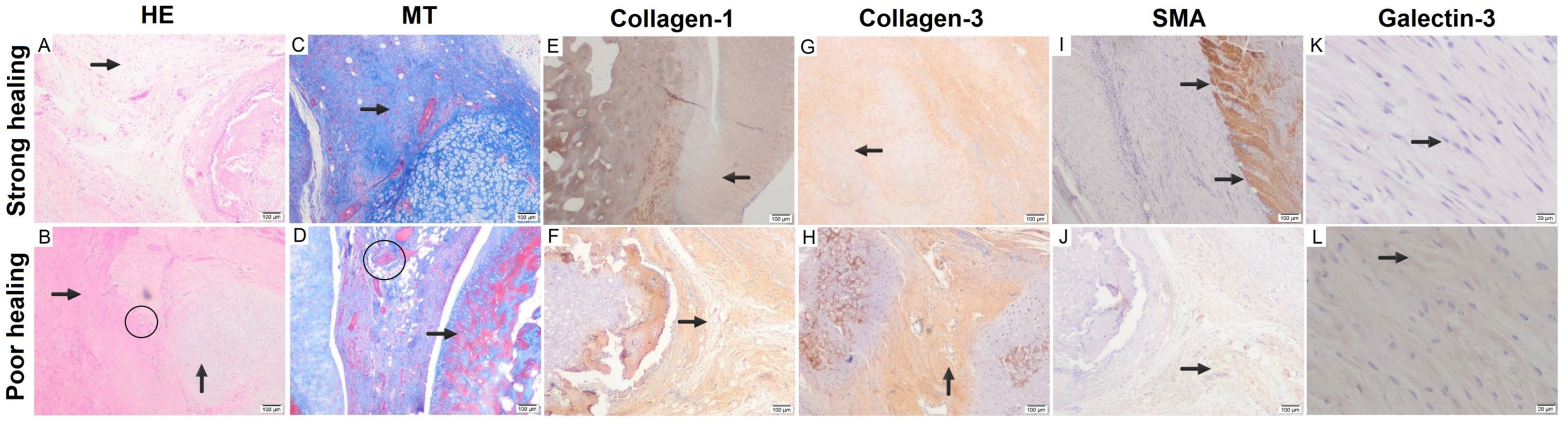
The key differences in histological changes between strong and poor healing conditions: **(A)** curled collagen structure, loose stromal structure, regular configuration (arrow), strong healing (HE, x100). **(B)** Disrupted configuration with hyalinized collagen bundles (horizontal arrow), irregular cartilaginous tissue (vertical arrow), thick vascular bundles (encircled) (HE, x100). **(C)** Collagen bundles containing small nuclei structures (arrow) (MT, x100). **(D)** Thick and irregular vascular clusters (encircled), hyalinized fibrotic areas (arrow), and disorganized, poor healing (MT, x100). **(E)** Regular configuration with normal collagen bundles (arrow) (IHC, x100). **(F)** Abnormal collagen structure with thick hyalinized areas (arrow) in irregular configuration (IHC, x100). **(G)** Near-normal healing characterized by thin-walled capillaries (arrow) (IHC, x100). **(H)** Poor healing characterized by thick-walled and irregularly distributed vascularization (arrow) (IHC, x100). **(I)** Clear boundaries with absence of muscle fibers between collagen bundles (arrows) in separated, strong healing (SMA IHC, x200). **(J)** Irregular configuration of muscle fibers (arrow) observed in tendon healing area of poor healing (SMA IHC, x200). **(K)** Intensely stained and regular fibroblasts with Galectin-3 (arrow) (Galectin-3 IHC, x200) (HE, Hematoxylin–Eosin; MT, Masson Trichrome; IHC, immunohistochemistry; SMA, alpha smooth muscle actin). **(L)** Sparsely stained and irregular fibroblasts with Galectin-3 (arrow).

The samples were dissected and kept in 10% formalin fixative solution for 3 days. Then, the tissues were left under running water overnight, and the next day they were passed through a series of 60, 70, 80, and 100% alcohol, respectively, and then taken into xylene. Tissues were embedded in paraffin to obtain thin sections, and 3 μm sections were taken with a microtome. After the sections were kept in an oven at 60°C overnight and deparaffinized, routine Hematoxylin–Eosin (HE) and Masson Trichrome (MT) staining was performed as the first step of the morphological evaluation. After staining, the tissues were photographed with an Olympus CX41 microscope (Olympus Corp., Tokyo, Japan) at 20, 40, and 100 magnifications. The most common and established system for evaluating pathological changes in tendon tissue, particularly utilized in animal experiments, is the Bonar scoring system (44–46). Tenocytes, ground substance, collagen, and vascularization were assessed using the Bonar scoring system, each out of four points. The Bonar scoring system utilizes a four-point scale; ‘0’ represents a normal tendon appearance, ‘1’ indicates mild abnormal tendon appearance (10–20% of total tissue), ‘2’ represents moderately abnormal appearance (over 20% of total tissue), and ‘3’ indicates a markedly abnormal appearance. Total scores were scored from 0 (normal tendon, strong healing) to 12 (most severe detectable pathology, poor healing) and are shown in Figures 2A–D.

### 2.3 Immunohistochemical analysis

Five micrometer thick sections taken from paraffin blocks with the help of a microtome were then placed on polylysine slides (Sigma, St Louis, MO, United States). After the dehydration stage, microwave application was made in citrate solution with a pH of 6 (Carlo Erba, 368,057). Sections were kept in 3% hydrogen peroxide (Dako, Glastrup, Denmark) to prevent endogenous peroxidase activity. After blocking with normal goat serum, the sections were incubated with primary antibodies, which are type I collagen (1:100, Sigma, United States), type III collagen (1:100, Sigma, United States), alpha smooth muscle actin (SMA) (1:100, RTU, Leica, BK), and Galectin-3 (1:100, RTU, Cell Marque, CA, United States). After the biotin-labeled secondary antibody (Abcam, United Kingdom) step, the tissues treated with the streptavidin-HRP kit (Santa Cruz, USA) were finally treated with 0.05% diaminobenzidine (Zymed Histostain Plus CA, United States). All washes during the staining process were performed using 1X phosphate-buffered saline (PBS, 10 mM phosphate, 137 mM NaCl, 2.7 mM KCl, pH 7.4), purchased from Sigma-Aldrich (St. Louis, MO, United States). Immunohistochemical staining results were evaluated with *H*-score. Staining ratio was graded semiquantitatively (0 = staining in less than 1% of cells, 1+ = staining in 1–10% of cells, 2+ = staining in 11–50% of cells, 3+ = staining in 51–80% of cells, 4+ = staining in more than 80% of cells). Staining intensity was also determined by the blind method (0 = no staining, 1 = pale, 2 = moderate, 3 = intense). Then, the total score was calculated with the formula “(1 + staining intensity/3) x staining ratio.” The highest marked immunohistochemical images for H-score are shown in Figures 2E–L.

### 2.4 Biomechanical evaluation

**Figure 3:**
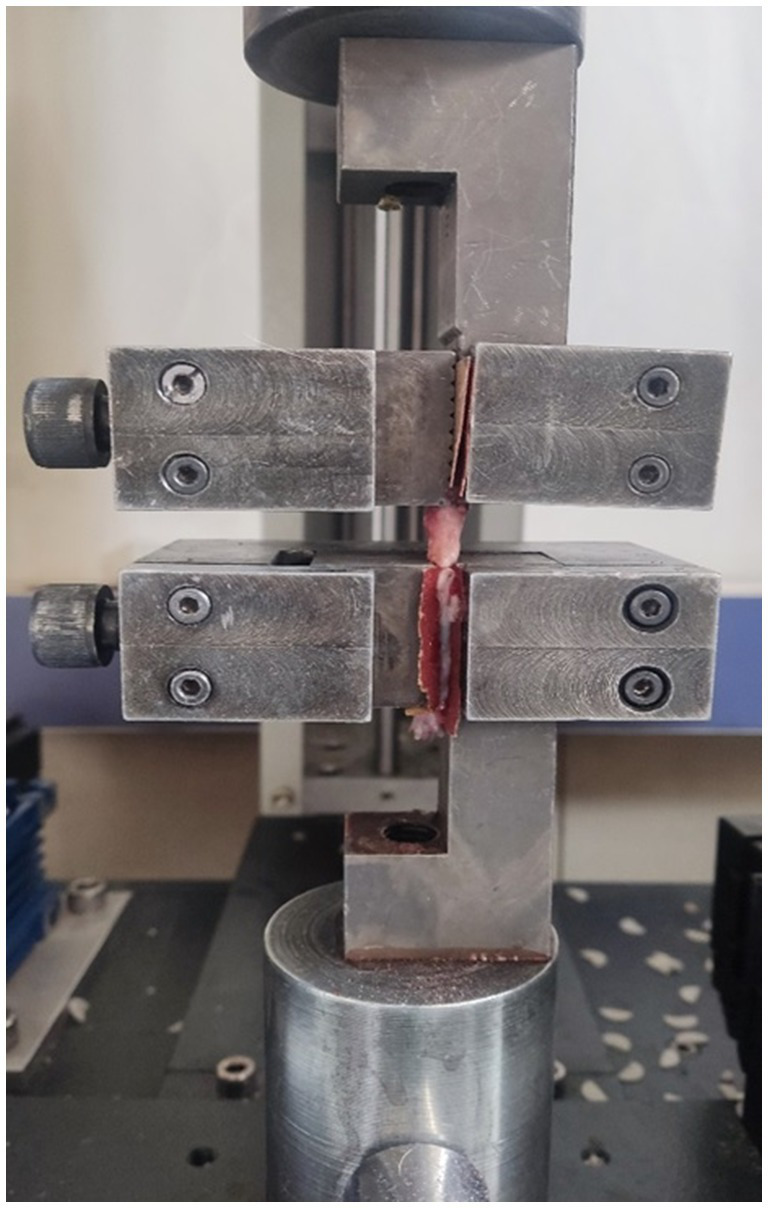
Application of tensile tear test to the Achilles tendons.

The transverse diameters of the rat’s Achilles tendons were measured using a digital caliper (TESA Unimaster, Renens, Switzerland) (47). It was assumed that the transverse area of the Achilles tendons followed circular geometry. To ensure tissue integrity, the tendons were kept moistened with gauze soaked in a physiological solution during preparation and mounting in the materials testing equipment. The proximal tendon was delicately separated from the muscle for clamping. The length of the tendon between the grips was estimated using a caliper. A tensile test was conducted on both the experimental and control groups’ Achilles tendons (total number of samples 32). Biomechanical tests were performed using the ELISTA, TST 2500 mxe brand axial tensile testing device. The tensile tests were executed under identical conditions for each Achilles tendon sample. Figure 3 illustrates the tensile tests, with the Achilles tendons secured between the jaws of the device, one end attached to the calcaneus and the other to the musculotendinous junction. A 5 kN load cell was employed to conduct the biomechanical tests on the specimens, fixed vertically in the machine using a fixture, at a crosshead speed of 1 mm/min at room temperature. Force (N) versus elongation (mm) was recorded during the tensile test. Parameters such as failure load (N), displacement (mm), total energy (J), ultimate stress (MPa), and strain (%) were derived from the force (N)-elongation (mm) graphs at the conclusion of the biomechanical tests.

### 2.5 Randomization and blinding

In this study, double-blinding and randomization strategies were implemented to reduce potential biases and enhance the reliability of the results. These double-blinding and randomization strategies were employed to enhance the internal validity of the study and enable the evaluation of results independently of external factors. The reliability of the obtained data and the scientific robustness of the study were supported by the integration of these methodological approaches.

The allocation of animals to groups was performed randomly using computer-based randomization methods. This helped balance potential differences among animals, initially while reducing the likelihood of systematic errors between experimental and control groups. In double-blinding, during the execution of the experiment, both the observers applying interventions to the animals and the individuals evaluating the data (microscopic preparations) were kept unaware of group assignments. This approach helped prevent potential biases in subjective assessments and data analysis.

### 2.6 Statistical analysis

SPSS 23 (Statistical Package for the Social Sciences) program was used for data analysis (Graph Pad Software, Inc., La Jolla, CA). Data were expressed as mean ± standard deviation (SD) or median (min–max). The skewness and kurtosis values of the data were observed to adhere to a normal distribution. Accordingly, an independent two-sample *t*-test (unpaired *t*-test) was utilized. A *p*-value of <0.05 was considered statistically significant.

## 3 Results

Throughout the experiment, no complications occurred in either rat group that would disrupt standardization or necessitate exclusion, such as death, wound site infections, or tendon rerupture. Additionally, no circumstances necessitating sample exclusion were observed within groups during data analysis.

### 3.1 Histopathological findings

**Figure 4:**
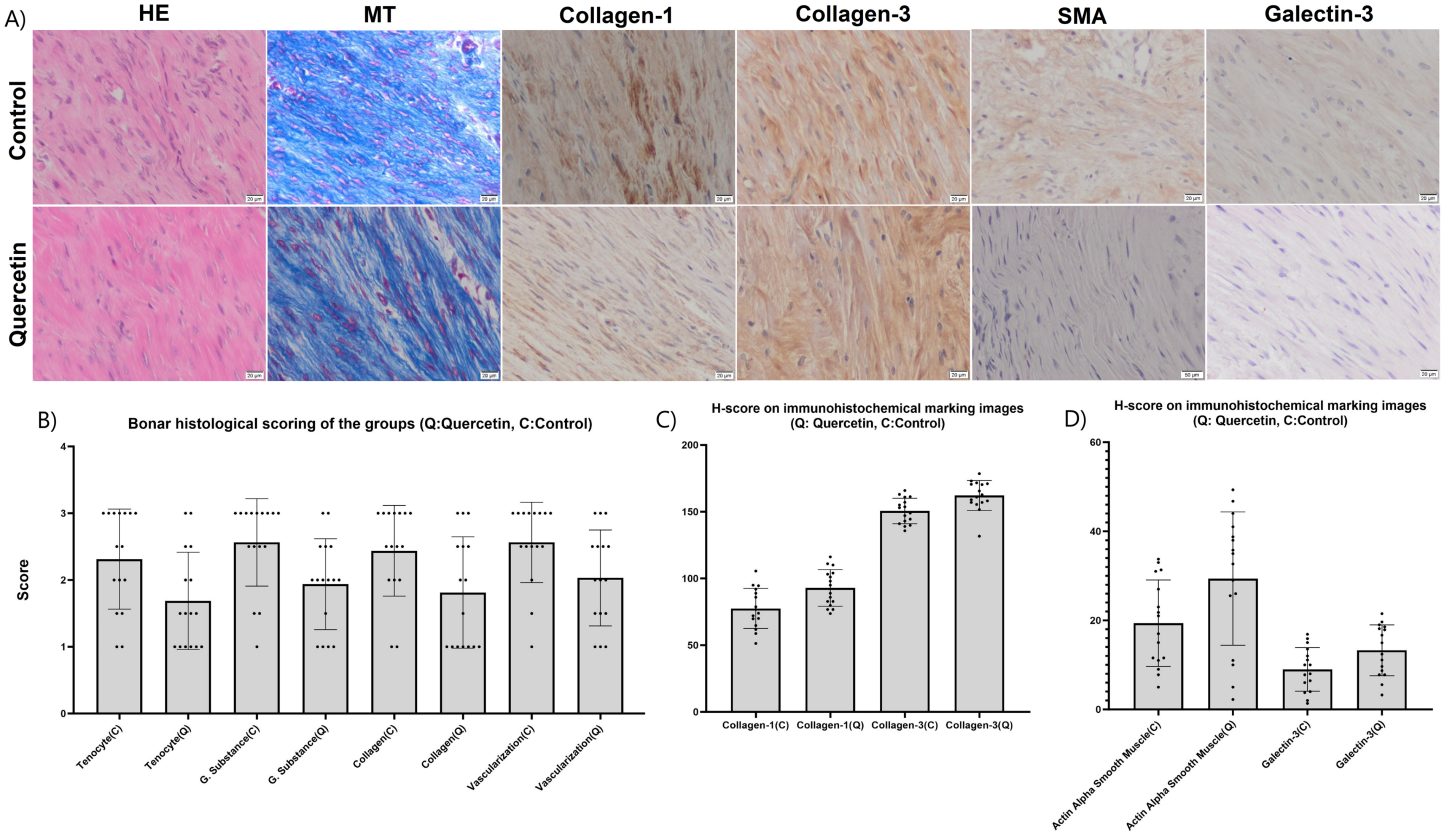
Visualization of microscopic image samples from control and quercetin groups. **(A)**. Graphical representation of comparison of control and quercetin groups according to Bonar histological scores **(B)**. Graphical representation of comparison of H-scores of control and quercetin groups according to Collagen-1 and Collagen-3 in immunohistochemical evaluation **(C)**. Graphical representation of comparison of H-scores of control and quercetin groups according to SMA and Galectin-3 in immunohistochemical evaluation **(D)**. (HE, Hematoxylin–Eosin; MT, Masson Trichrome; SMA, Alpha smooth muscle actin).

During the phases of tendon healing, structures typically observed microscopically, such as tenoblasts, inflammatory cell infiltration, collagen fibrils, and extracellular matrix, were identified in both the experimental and control groups. It was observed that tenoblasts lost their elongated appearance and alignment, while the density of extracellular matrix increased, and tenoblasts decreased in size. Inflammatory cells, newly formed collagen bundles, mature collagen bundles, and tenocytes starting to line up in wavy patterns were observed and depicted as shown in Figures 4A,B. According to the Bonar scoring, tenocytes were 2.31 ± 0.75 in the control group and 1.69 ± 0.73 in the quercetin group, indicating a significant difference between them (*p* = 0.0232). Ground substance was 2.56 ± 0.66 in the control group and 1.93 ± 0.68 in the quercetin group, showing a significant difference between them (*p* = 0.0128). Collagen was 2.44 ± 0.68 in the control group and 1.81 ± 0.83 in the quercetin group, indicating a significant difference between them (*p* = 0.0272). Vascularization was 2.44 ± 0.68 in the control group and 1.81 ± 0.83 in the quercetin group, showing a significant difference between them (*p* = 0.0307). As shown in Table 1, morphometric scoring revealed significant differences between the control and experimental groups in terms of parameters.

**Table 1:** Tenocyte morphology and proliferation, presence or absence of ground substance, collagen bundle properties, vascularity were evaluated using Bonar scoring system in Hematoxylin–Eosin and Masson Trichrome staining.

	Control group (*n*:16)		Quercetin group (n:16)		
	Mean ± SD	Min–Max–Med	Mean ± SD	Min–Max–Med	*p*
Tenocytes	2.31 ± 0.75	1–3–2.5	1.69 ± 0.73	1–3–2	0.0232*
Ground substance	2.56 ± 0.66	1–3-3	1.93 ± 0.68	1–3–2	0.0128*
Collagen	2.44 ± 0.68	1–3–3	1.81 ± 0.83	1–3–2	0.0272*
Vascularization	2.56 ± 0.60	1–3–3	2.03 ± 0.72	1–3–2	0.0307*

Scoring was made between 0 (best) and 3 (worst) (*p*: unpaired *t*-test, *significant *p*-value, written in bold).

### 3.2 Immunohistochemical findings

In immunohistochemical analyses, tendon healing and ground substance were evaluated using type I collagen, type III collagen, and SMA. To evaluate the relationship between Galectin-3 and fibrosis, Galectin-3 was applied using immunohistochemical methods. The evaluation of Galectin-3 involved counting stained fibroblastic cells under a light microscope at X400 magnification. Following staining with Galectin-3, fibroblasts were differentiated from monocytes, macrophages, and neutrophil cells, and the morphological characteristics of fibroblasts were analyzed in detail. In this analysis, the number of fibroblasts exposed to Galectin-3 was determined, and morphological features associated with fibrosis were identified. As shown in Figure 4A, the region with the highest number of fibroblasts was selected as the focal point of this evaluation.

As shown in Figure 4A, areas with the highest cell count were preferred. In H-score evaluations, Collagen-1 was 78.03 ± 15.17 in the control group and 91.15 ± 14.59 in the quercetin group, indicating a significant difference between them (*p* = 0.0166). Collagen-3 was 150.54 ± 9.53 in the control group and 162.13 ± 11.17 in the quercetin group, showing a significant difference between them (*p* = 0.0166). SMA was 19.36 ± 9.78 in the control group and 29.38 ± 14.99 in the quercetin group, indicating a significant difference between them (*p* = 0.0323). Galectin-3 was 8.99 ± 4.90 in the control group and 13.29 ± 5.71 in the quercetin group, showing a significant difference between them (*p* = 0.0295). All immunohistochemical parameters are provided in Table 2 and Figures 4C,D.

**Table 2:** COLI, COLIII, SMA, and GLT evaluation with *H*-score on immunohistochemical marking images.

	Control group (*n*:16)		Quercetin group (*n*:16)		
	Mean ± SD	Min–Max Med	Mean ± SD	Min–Max Med	*p*
COLI	78.03 ± 15.17	55.65–111.65 77.44	91.15 ± 14.59	71.77–115.01 85.93	0.0166*
COLIII	150.54 ± 9.53	135.67–165.89 151.01	162.13 ± 11.17	131.67–178.54 161.68	0.0036*
SMA	19.36 ± 9.78	5.01–33.76 19.01	29.38 ± 14.99	2.24–49.33 33.83	0.0323*
GLT	8.99 ± 4.90	1.35–16.88 8.99	13.29 ± 5.71	3.781–21.111 13.39	0.0295*

COLI, Type I collagen; COLIII, Type III collagen; SMA, alpha smooth muscle actin; GLT, Galectin-3 (*p*: unpaired *t*-test, *significant *p*-value, written in bold).

### 3.3 Biomechanical findings

**Figure 5:**
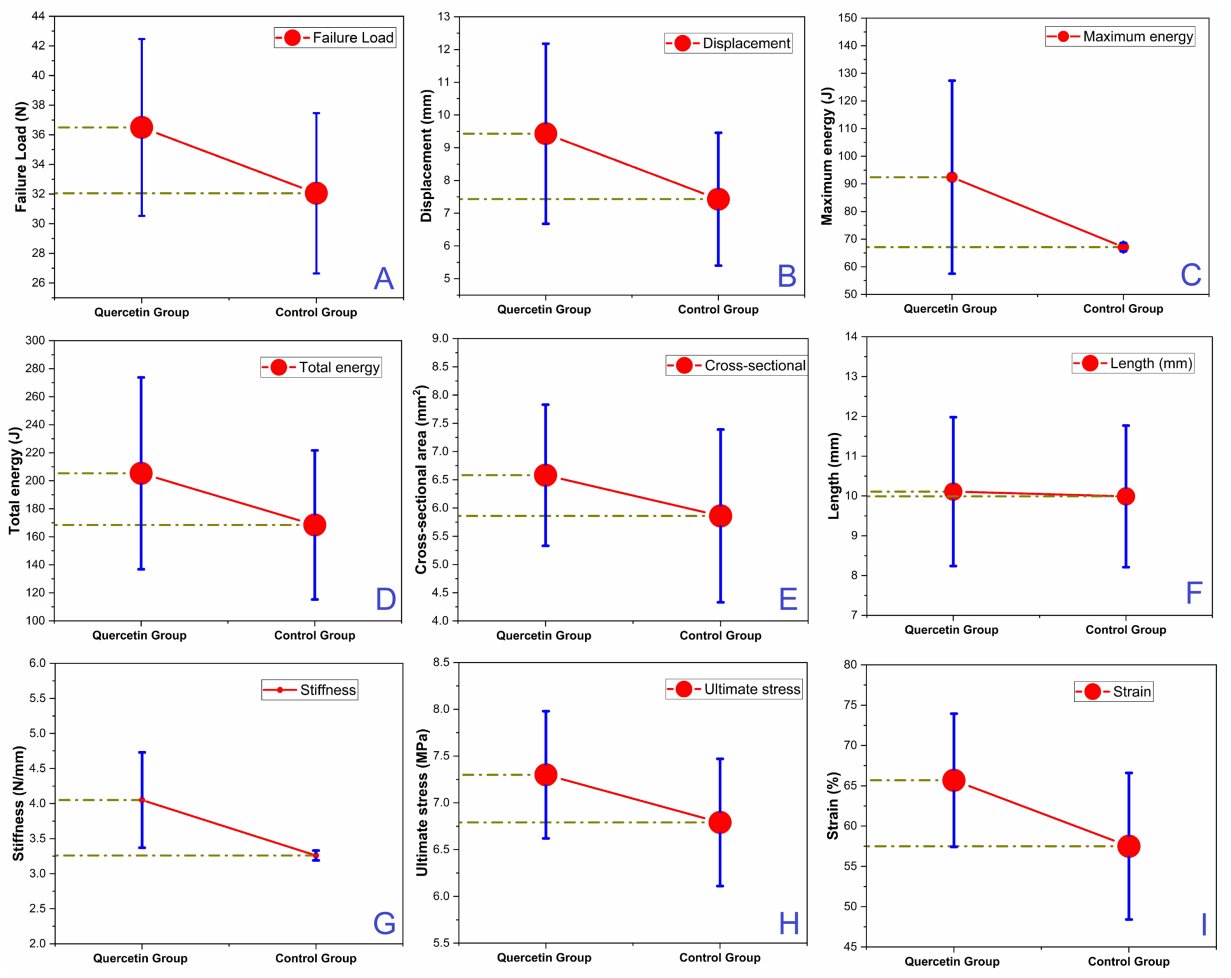
The comparison of biomechanical parameters in both groups: **(A)** failure load (N), **(B)** displacement (mm), **(C)** maximum energy (J), **(D)** Total energy (J), **(E)** Cross-sectional area (mm^2^), **(F)** Length (mm), **(G)** Stiffness (N/mm), **(H)** Ultimate stress (MPa), **(I)** Strain (%).

The biomechanical data regarding the resistance against applied loads of Achilles tendons utilized in this study are presented in Table 3. Biomechanical test outcomes of Achilles tendons for both the quercetin and control groups are depicted in Figure 5. Analysis of failure load (N), displacement (mm), total energy (J), ultimate stress (MPa), and strain (%) data derived from the biomechanical tests for both quercetin and control group samples was conducted in detail. Initially, upon examining the average failure load, the quercetin group’s results (36.5 N) demonstrated mechanically superior outcomes compared to the control group (32.06 N) by approximately 12% (Figure 5A). Displacement and strain values exhibited greater elongation in the quercetin group, suggesting that Achilles tendons exhibit higher elongation under mechanical load in this group (Figures 5B,I). In terms of total energy, the calculation was automatically derived from the program of the testing device, representing the area under the force-strain graph. The total energy (J) and maximum energy (J) values were found to be approximately 18 and 27% higher, respectively, in the quercetin group test results compared to the control group (Figures 5C,D). One of the most critical mechanical parameters in biomechanical tests is ultimate stress. The results of this study indicated that the average ultimate stress values of the quercetin group samples were approximately 7% higher than those of the control group (Figure 5H). Stiffness refers to the resistance of a specimen against deformation when subjected to a mechanical force. It is determined by the gradient of the force-strain curve within the ‘linear’ zone. In the present study, stiffness value was calculated to be approximately 20% higher in the quercetin group compared to the control group (Figure 5G). This outcome suggests a positive healing effect of quercetin in the experimental group.

**Table 3:** Comparison of biomechanical parameters in both groups.

	Control group (*n*:16)		Quercetin group (*n*:16)		
	Mean ± SD	Min–Max Med	Mean ± SD	Min–Max Med	*p*
Failure load (N)	32.06 ± 5.14	24–41 32	36.5 ± 5.97	26–45 38	0.032*
Displacement (mm)	7.43 ± 2.03	4–11 8	9.43 ± 2.75	5–14 9.5	0.026*
Maximum energy (J)	67.12 ± 13.14	41–85 72.5	92.43 ± 34.93	45–142 93	0.014*
Total energy (J)	168.5 ± 53.20	96–241 169.5	205.31 ± 68.47	106–294 198.5	0.1
Cross-sectional area (mm^2^)	5.86 ± 1.53	3.8–8.1 5.6	6.58 ± 1.25	4.8–8.1 6.6	0.161*
Length (mm)	9.99 ± 1.78	7.1–11.9 10.75	10.11 ± 1.87	7.5–13.1 10.15	**0.848**
Stiffness (N/mm)	3.26 ± 0.07	2.4–4.9 3.25	4.05 ± 1.12	2.5–5.6 3.75	0.025*
Ultimate stress (MPa)	6.79 ± 0.68	6.10–7.90 6.50	7.30 ± 0.68	6.10–8.10 7.55	0.045*
Strain (%)	57.5 ± 9.09	48–70 56	65.68 ± 8.27	53–78 65.5	0.012*

N, Newton; mm, milimeter; J, Joule; MPa, megapascal (*p*: unpaired *t*-test, *significant *p*-value, written in bold).

## 4 Discussion

Quercetin remains a subject of ongoing research interest due to its diverse biological functions, including antioxidative, anti-inflammatory, and anticarcinogenic properties, among others. However, upon conducting our literature review, we identified a notable gap: there is currently no study investigating the healing effects of quercetin in a tendon rupture model. Building upon this observation, the primary aim of our study was to assess the impact of quercetin on tendon healing, filling this gap in the existing literature. Through this investigation, we sought to contribute valuable insights into the potential therapeutic role of quercetin in tendon injuries.

Tendon injuries are traumas frequently encountered by orthopedic surgeons that cause individual pain, disability, and institutional health expenditures (47). Although significant progress has been made in the treatment of tendon injuries with the advancing technology and developments in medicine, unsatisfactory results are still encountered in some patients. It is necessary to search for agents that can be obtained from natural products such as quercetin, which will increase tendon regeneration (8).

In our study, we utilized a total Achilles tendon injury model to investigate the healing effects of oral quercetin on this tendon. We evaluated four different histochemical parameters (tenocyte, collagen, vascularity, and ground substance) comprising the Bonar scoring system, as well as four different immunohistochemical parameters (type I collagen, type III collagen, SMA, and Galectin-3). The results indicated that all these parameters were significantly higher in the quercetin group compared to the control group. Furthermore, biomechanical tests were conducted wherein the Achilles tendons were subjected to tearing from the repaired site. Among the six biomechanical parameters measured, the quercetin group exhibited significantly higher values than the control group. Notably, there was no significant difference between the two groups in the remaining three parameters. Of particular importance, ‘failure load’ and ‘tendon rupture displacement,’ which are crucial clinical indicators, were found to be significantly higher in the quercetin group. The significant improvements observed in these biomechanical parameters, along with the histopathological and immunohistochemical data, suggest that quercetin supplementation may facilitate early mobilization, improve range of motion, and reduce adhesion formation. These findings highlight the potential therapeutic benefits of quercetin in promoting tendon healing and enhancing functional outcomes.

In their study on mice, Siddiqi and Zargar stated that quercetin led to an increase in collagen and hydroxyproline levels in the liver (48). Liang et al. (10) evaluated tendon adhesion after Achilles tendon injury in rats. They found that tendon adhesion was less in rats given quercetin. They reported that the reason for this is that quercetin, which is a powerful antioxidant, reduces the effectiveness of reactive oxygen species, which are the main mediators in fibrogenesis. In our study, we conducted Galectin-3 immunohistochemical staining to evaluate fibrogenesis, and we found a significant increase in the quercetin group compared to the control group. This indicates that quercetin inhibits fibrosis in tendon healing, which is supported by Liang et al.’s study. The higher levels of collagen in the quercetin group and the higher force required for tendon rupture in biomechanical tests may suggest tissue regeneration instead of fibrosis. Both Liang et al.’s study and our study strengthen the hypothesis that quercetin has positive effects on tendon healing due to its antioxidative properties.

Quercetin is found in the traditional Chinese herbal medicine *Hippophae rhamnoides*, along with isorhamnetin and kaempferol. It is known that this herbal medicine is used in the treatment of tendon injuries in Far Eastern societies (49, 50). Fu et al. (7) evaluated the effect of *Hippophae rhamnoides* in rats with a patellar tendon injury model. They stated that it supports the ultimate stress in patellar tendon healing by positively affecting the cytokine profile and matrix deposition in the tendon regeneration region, but further studies should be done due to the presence of more than one component in *Hippophae rhamnoides*. We investigated the effect of quercetin, one of the components of *Hippophae rhamnoides*, on tendon healing and found that quercetin increased ultimate stress in the healing of the Achilles tendon. We believe that these natural products, which are widely used in the Far East, should be given more place in the modern medical literature.

A clinical phase-1 study has reported that quercetin inhibits tyrosine kinases, which are enzymatic receptors involved in cell signaling pathways, and exhibits antineoplastic properties. In phase-2 trials, bolus intravenous administration of 2.5 g/70 kg quercetin at weekly or three-week intervals has been recommended (51, 52). Additionally, studies have suggested the use of 500 mg/day quercetin supplementation, administered twice daily for 1 month, in the treatment of prostatitis and interstitial cystitis (53, 54). We think that similar clinical studies focusing on musculoskeletal diseases should also be conducted in the future, as quercetin might yield promising therapeutic results in this area. However, prior investigations focusing on the signaling pathways involved in Achilles tendon healing, including comprehensive *in vivo* and *in vitro* studies using various animal models, are essential to provide a solid foundation for subsequent clinical trials.

The most important limitation of our study was the lack of literature information to compare our study, since no animal studies or human clinical trials on tendon healing of quercetin have been conducted before. Another limiting aspect of the study is the lack of quantitative molecular analyses of quercetin in both *in vivo* and *in vitro* settings. However, given our current knowledge, this study, being the first to examine the effect of quercetin on tendon healing, has yielded promising results for future molecular-level investigations.

## 5 Conclusion

It was observed that quercetin, which is easily accessible due to its widespread availability in nature and low cost, significantly increased tendon healing both biomechanically and immunohistochemically. However, since this study is the first to examine the role of quercetin in tendon healing, further investigations, including comprehensive *in vivo* and *in vitro* studies on the underlying mechanisms and signaling pathways involved in tendon healing, are necessary to establish a solid foundation before clinical studies can be conducted to evaluate its effects on humans.

## Data Availability

The raw data supporting the conclusions of this article will be made available by the authors, without undue reservation.
